# Nanostructures for Photonics and Optoelectronics

**DOI:** 10.3390/nano12111820

**Published:** 2022-05-26

**Authors:** Vicente Torres-Costa

**Affiliations:** Departamento de Física Aplicada, Universidad Autónoma de Madrid, Cantoblanco, 28049 Madrid, Spain; vicente.torres@uam.es

As microelectronic technology approaches the limit of what can be achieved in terms of speed and integration level, there is an increasing interest in moving from electronics to photonics, where photons and light beams replace electrons and electrical currents, which will result in higher processing speeds and lower power consumption. In the meantime, advanced optoelectronic devices bridge the gap between these two technologies. While optoelectronics deals with the integration of optics into electronics, photonics fully considers optical devices.

In this new technology, nanostructures play a key role. In contrast to nanomaterials, whose properties are inherent, nanostructures are purposedly engineered to present unique optical and photonic properties by taking advantage of phenomena such as quantum confinement effects, localized plasmons, and interference or effective media properties. Bottom-up nanostructures (epitaxial layers, quantum dots and wires, and bidimensional materials) and up-bottom materials (meso- and nanoporous semiconductors and meta structures) can be tailored to demonstrate the appropriate behavior of a given application.

This Special Issue of *Nanomaterials* offers readers a compilation of some of the latest research in the field of the design, fabrication, characterization, and application of nanostructures in different fields of science. Articles published herein describe the fabrication of novel structures, such as the patterned metasurfaces developed by D. Huo et al. [1] or the decoration of TiO_2_ 1D nanostructures with ZnO nanofilaments [2], and their characterization [3]. However, this Special Issue also aims to provide an overview of the applications of these nanostructures in different areas, such as the highly interesting gold nanostars used for biosensing developed by A. Tukova and colleagues [4], or the photonic applications of infiltrating nanostructured porous silicon with silver nanoparticles, a sort of nano-in-nano approach [5].

Ultimately, we hope this Special Issue is interesting and helpful for researchers working in the field of nanostructures, students and the casual reader. Finally, I would like to express my sincere gratitude to the authors and their colleagues who have contributed to this Special Issue of *Nanomaterials*.
